# Genotypic Diversity among Angolan Children with Sickle Cell Anemia

**DOI:** 10.3390/ijerph18105417

**Published:** 2021-05-19

**Authors:** Mariana Delgadinho, Catarina Ginete, Brígida Santos, Armandina Miranda, Miguel Brito

**Affiliations:** 1 H&TRC—Health & Technology Research Center, ESTeSL—Escola Superior de Tecnologia da Saúde, Instituto Politécnico de Lisboa, 1990-096 Lisbon, Portugal; mariana.delgadinho@estesl.ipl.pt (M.D.); catarina.ginete@estesl.ipl.pt (C.G.); 2 Centro de Investigação em Saúde de Angola (CISA), Caxito, Angola; santosbrigida@yahoo.com.br; 3 Hospital Pediátrico David Bernardino (HPDB), Luanda 3067, Angola; 4 Instituto Nacional de Saúde Doutor Ricardo Jorge (INSA), 1649-016 Lisbon, Portugal; armandina.miranda@insa.min-saude.pt

**Keywords:** sickle cell anemia, fetal hemoglobin, HBB haplotypes, BCL11A, BGLT3, HBG2, HBS1L-MYB, NGS

## Abstract

Background. Sickle cell anemia (SCA) is an inherited blood disorder that affects over 300,000 newborns worldwide every year, being particularly prevalent in Sub-Saharan Africa. Despite being a monogenic disease, SCA shows a remarkably high clinical heterogeneity. Several studies have already demonstrated the existence of some polymorphisms that can provide major clinical benefits, producing a mild phenotype. Moreover, the existence of distinct haplotypes can also influence the phenotype patterns of certain populations, leading to different clinical manifestations. Our aim was to assess the association between polymorphisms in genes previously related to SCA disease severity in an Angolan pediatric population. Methods. This study analyzed clinical and biological data collected from 192 Angolan children. Using NGS data, we classified the HBB haplotypes based on four previously described SNPs (rs3834466, rs28440105, rs10128556, and rs968857) and the genotype for the SNPs in HBG2 (rs7482144), BCL11A (rs4671393, rs11886868, rs1427407, rs7557939), HBS1L-MYB (rs66650371) and BGLT3 (rs7924684) genes. Results. The CAR haplotype was undoubtedly the most common HBB haplotype in our population. The HbF values and the ratio of gamma chains were statistically significant for almost all of the variants studied. We reported for the first time an association between rs7924684 in the BGLT3 gene and gamma chains ratio. Conclusions. The current findings emphasize the importance personalized medicine would have if applied to SCA patient care, since some of the variants studied might predict the phenotype and the overall response to treatment.

## 1. Introduction

Sickle cell anemia (SCA) is an inherited and life-threatening blood disorder that affects over 300,000 newborns worldwide every year [1,2]. It is particularly common in sub-Saharan Africa, with around 75% of SCA births occurring in this region [2,3]. Although there are limited accurate data available on the true SCA mortality in Africa, some studies conclude that this widely neglected disease has an extremely high mortality rate between 50–90% in undiagnosed children under five [4,5]. Some patients can suffer from osteonecrosis, strokes, multiple pain crises and usually require frequent hospitalizations, while others only have mild anemia and may not suffer from any complications for decades [6,7]. Despite being a monogenic disease, SCA shows a remarkably high clinical heterogeneity [8].

One of the first modifiers of disease severity to be discovered was the fetal hemoglobin (HbF), which is a strongly heritable trait [6,9]. Normally, it is less than 1% in nonanemic persons, but these levels can vary considerably in an SCA patient and can be as high as 25%, producing a mild phenotype [10,11]. Higher HbF levels can provide major clinical benefits, being associated with a reduced rate of acute painful episodes, fewer osteonecrosis events, rare acute chest syndromes, fewer leg ulcers, and reduced disease severity [12]. 

Several studies have already demonstrated that HbF levels are regulated by variants in three main quantitative trait loci (QTL): BCL11A, HBS1L-MYB intergenic region, and Xmn1-HBG2, which together can contribute to between 20–50% of HbF variability in SCA patients [11,13,14]. More specifically, it was proven by the use of a multiple linear regression approach, that the combination of only three variants in those three regions (rs1427407, rs66650371 and rs7482144) could account for 31.9% of HbF variance [15].

To date, SNPs within the intron 2 of BCL11A appear to have the most impact on HbF expression [16]. However, new variants are being uncovered with genome-wide association studies, and the challenge is now to correlate them with disease severity in different populations.

Due to the protective effect in malaria, the sickle mutation spreads to different geographical regions, and haplotypes surrounding this mutation have subsequently diversified, leading to the phenotype patterns observable today [6,17]. Genetic analysis of the HBB gene cluster has revealed five distinct HbS haplotypes: Senegal (SEN), Benin (BEN), Bantu or Central African Republic (CAR), Cameroon (CAM), and Arab-Indian (AI) [18,19]. It has been shown that patients with AI or SEN haplotypes have the highest HbF levels associated with fewer clinical manifestations, and this is likely due to a higher prevalence of the Xmn1-HBG2 polymorphism [6,14]. BEN and CAM haplotypes exhibit an intermediate disease severity [20]. Meanwhile, patients with CAR haplotypes have the lowest HbF levels, and therefore the worst clinical course [21]. 

The aim of this study was to assess the frequency and the influence of polymorphisms in the SCA severity of an Angolan population. Specifically, we intend to analyze SNPs in the β-globin gene cluster, BCL11A gene, and characterize HbS haplotypes. Understanding the clinical heterogeneity could provide valuable insights into how such variants could be used as prognostic markers, create opportunities for personalized medicine, and could even lead to the development of new targets for future treatments [22].

## 2. Materials and Methods

### 2.1. Study Population

The sample population consisted of 192 SCA Angolan children selected from a cohort from the Hospital Pediátrico David Bernardino and the Centro de Investigação em Saúde de Angola (CISA) at Hospital Geral do Bengo. As for the eligibility criteria, none of the patients were treated with hydroxyurea or had a blood transfusion in the last three months prior to data collection. In the first medical appointment, a full anamnesis questionnaire was obtained, including social and family context, previous manifestations of the disease, and the current symptoms. A neurological and physical exam was also performed.

This study was approved by the Ethical Committee of Ministry of Health of Angola (CE. N° 040/2018), and the Ethical Committee of ESTeSL (CE-ESTeSL-N°.43-2018). Informed consent was obtained and signed by the children’s caregivers. All the consultations and follow-ups were performed by the project team freely, and with all the human research standards adopted in the Helsinki declaration.

### 2.2. Hematological and Biochemical Parameters

At the routine medical follow-up consultations, each patient was subject to clinical examination by a specialized pediatrician and collection of a whole blood sample, used for hematological analyses, electrophoresis for SCA confirmation, and fetal hemoglobin quantification. The hematological parameters measured were the following: complete blood count (erythrocytes reticulocytes, white blood cells and platelets), hemoglobin, mean corpuscular volume (MCV), mean corpuscular hemoglobin (MCH) using the XT-2000i Hematology Analyzer (Sysmex Corporation, Kobe, Japan). The hemoglobin fractions, including HbF, were quantified by HPLC (Biorad Variant II, Hercules, CA, USA). The relative quantification of individual globin chains G gamma and A gamma chains was carried out by reversed-phase HPLC (Agilent 1100, Soquimica, Lisboa, Portugal).

Biochemical blood tests were performed to complement the evaluation of the patient’s overall status. Lactate dehydrogenase (LDH), creatinine, urea, total and direct bilirubin, Aspartate Aminotransferase (AST) and Alanine Aminotransferase (ALT) levels were determined using Cobas C111 (Roche Diagnostics, Basel, Switzerland) and Mindray BA-88A (Mindray, Shenzhen, China).

### 2.3. Sequencing Analysis

Genomic DNA was extracted and purified from peripheral blood samples using the QIAamp DNA Blood Mini Kit (Qiagen GmbH, Hilden, Germany) according to the manufacturer’s recommendations. In order to achieve an ideal concentration for DNA sequencing-library preparation, all samples were quantified by Qubit™ dsDNA HS fluorometric assay (ThermoFisher Scientific Inc., Waltham, MA, USA) and normalized to a 100 ng concentration. A custom enrichment panel was designed for regions of interest in chromosomes 2, 6, 7, 11, 16, and 19. Sequencing libraries were constructed using the Illumina Nextera Flex for Enrichment and dual index barcodes from the IDT for Illumina UD Indexes Set A. The amplified fragments were purified using AMPure XP magnetic beads (BeckmanCoulter, Brea, CA, USA) and library quality was determined using the High Sensitivity D1000 reagents on an Agilent 4200 TapeStation System. Paired-end sequencing was performed on a NextSeq 550 sequencer (Illumina, San Diego, CA, USA) using the NextSeq 500/550 Mid-Output kit v2 (300 cycles). Samples were aligned with the reference GRCh37/hg19 human genome and variant analysis was performed using the Illumina Variant Studio V.3.0.

### 2.4. Haplotype Analysis 

**Figure 1:**
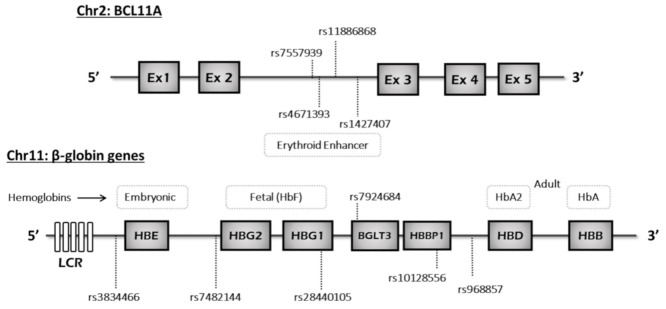
β-globin cluster and BCL11A gene representation. All the SNPs were identified through NGS analysis and four of these were used in haplotype classification.

HbS haplotype classification was based on four previously identified SNPs (rs3834466, rs28440105, rs10128556, and rs968857) [23], located in the β-globin gene cluster (Figure 1). These SNPs were estimated for the 192 SCA individuals of this study in order to define the five common haplotypes (BEN, CAR, SEN, CAM and AI). For each haplotype, the average HbF level and other clinical parameters were compared and calculated using SPSS version 26. Linkage disequilibrium and Hardy–Weinberg equilibrium were determined using GENEPOP 24. Additionally, we used the Arlequin software (version 3.5.2.2, Swiss Institute of Bioinformatics, 1015 Lausanne, Switzerland) to define haplotype blocks for the BCL11A polymorphisms.

### 2.5. Statistical Analysis

Hardy–Weinberg equilibrium and Linkage disequilibrium and were determined using GENEPOP [24]. Arlequin software version 3.5.2.2 [25] (Swiss Institute of Bioinformatics, 1015 Lausanne, Switzerland) was used to define haplotype blocks for the BCL11A polymorphisms. The normal distribution of the quantitative variables was verified by the Kolmogorov–Smirnov or Shapiro–Wilk test. Statistical significance for comparing hematological and biochemical data between haplotypes was performed using a non-parametric Mann–Whitney U test for single comparisons and Kruskal–Wallis for more than two groups. One-way ANOVA analysis was used for multiple group comparisons between the different polymorphisms. All statistical analysis was performed using the SPSS software version 26 (IBM Corp, Armonk, NY, USA) and *p*-values less than 0.05 were considered significant. Results for numeric variants were expressed as mean ± standard deviation.

## 3. Results

### 3.1. Clinical Findings

The patients were comprised of both genders (51.6% females) and they ranged in age from 3 to 12 years old (mean of 6.6 ± 2.5 years). The average age for SCA diagnosis was 35.8 months and this range was highly variable (3 to 144 months). Although 70.3% of patients experienced symptoms before completing one year, only half of these symptomatic children (52.6%) were medically diagnosed in the first year. The mean age for first symptoms manifestation was 14.3 months, where dactylitis was the most common symptom (69.3%), followed by pain episodes (22.4%) and severe anemia (7.8%). The number of hospitalizations varied between 0 to 22 events (mean of 2.9 events) and 16.7% of the sampled population was never hospitalized. 28.6% of the children had never received a transfusion and the mean was 2.4 transfusions. The percentual values of HbF ranged between 0.7 and 23.8% (mean of 5.65 ± 3.98 %) and total hemoglobin levels ranged from 4.25 to 10.00 g/dL (mean of 7.33 ± 0.97 g/dL). Table 1 and Table 2 summarize the clinical characteristics of the patients according to their genotype.

### 3.2. Genetic Findings

**Figure 2:**
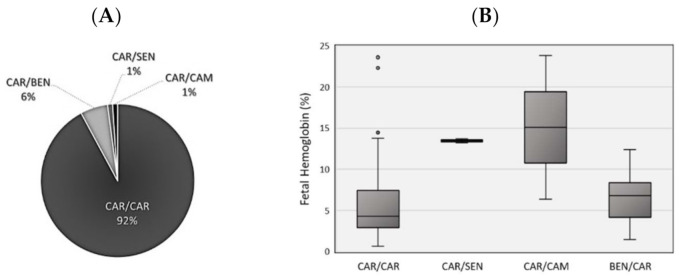
(**A**) Frequency of HBB haplotypes in the studied SCA population; (**B**) Distribution of HbF levels at different haplotype groups. Lines of the boxes represent the lower quartile, median, and upper quartile, and the outliers are the points outside the whiskers.

The distribution analysis of haplotype frequencies (Figure 2A), showed that the most prevalent was the homozygous CAR/CAR, detected in 92.15% (*n* = 176), followed by the CAR/BEN in 5.76% (*n* = 11). The other haplotypes observed were very rare: 1.05% CAR/SEN (*n* = 2), 1.05% CAR/CAM (*n* = 2). The statistical analysis showed significant differences between groups for the HbF values (Table 1). In the sampled population, the CAR/CAM individuals had the higher values of erythrocytes, HbF (Figure 2B) and hemoglobin, whereas the CAR/CAR had the lowest. As for the gamma ratio parameter, the CAR/CAR had the lowest values and the CAR/SEN the highest.

**Figure 3:**
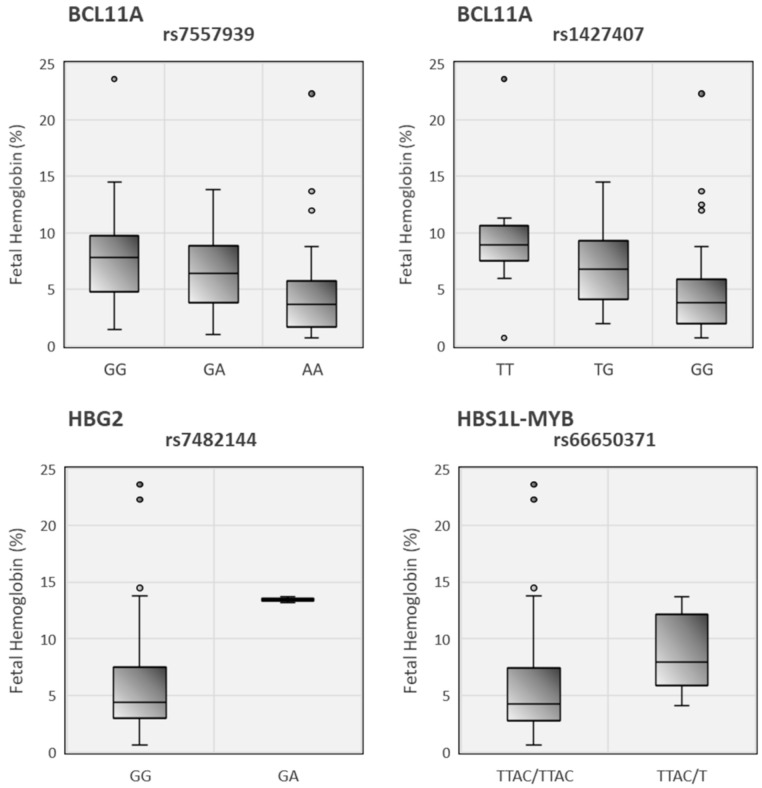
Distribution of HbF levels at different SNP genotypes in the three main QTL. Lines of the boxes represent the lower quartile, median, and upper quartile, and the outliers are the points outside the whiskers. *p*-values are shown in Table 2.

Additionally, we explore the frequency of some relevant polymorphisms already described in the literature: rs4671393, rs11886868, rs1427407, rs7557939 in BCL11A gene, rs66650371 in HBS1L-MYB intergenic region, rs7482144 in Xmn1-HBG2 and rs7924684 in BGLT3 gene (Table 2). The HbF values were statistically significant for almost all of the variants studied, except for rs7924684 (*p* = 0.053). Five of the SNPs had a significant association with the ratio of gamma chains. The genotypes of the polymorphisms in BCL11A gene were statistically associated with fetal hemoglobin (Figure 3). And three of them were significantly associated with neutrophil count and gamma chain ratio.

**Figure 4:**
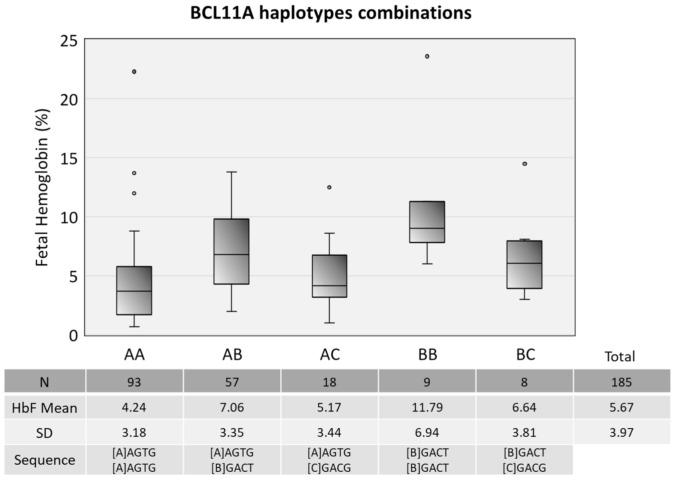
Distribution of HbF levels in different combinations of haplotypes made with four SNPs in BCL11A gene in the following order: rs7557939, rs4671393, rs11886868 and rs1427407. The Arlequin software defined ten different combinations using these four polymorphisms from the study’s cohort. Due to the low frequency of some combinations, this table only includes the five most prevalent. Lines of the boxes represent the lower quartile, median, and upper quartile, and the outliers are the points outside the whiskers. Statistical analysis showed a significant difference of the mean HbF value within the five groups (*p*-value < 0.001) by the one-way ANOVA.

All of the studied polymorphisms were in Hardy–Weinberg equilibrium (*p* > 0.05). We defined a linkage disequilibrium region in chromosome 2, including the BCL11A SNPs. The Arlequin software defined ten different combinations of haplotypes in our cohort, but given the low frequency of some, we only included in this paper the most prevalent ones. These genotypes are compared with HbF values in Figure 4.

## 4. Discussion

The clinical heterogeneity observed in SCA patients still presents a challenge in patient management. Some individuals can present nearly normal hemoglobin levels, being clinically asymptomatic, while others suffer from frequent and extremely severe pain crises, acute clinical events, and early mortality [12,24]. This highlights the importance of conducting studies to better understand the polymorphisms that contribute to this clinical outcome disparity.

In this study, we have analyzed the effect of HBB haplotypes and seven common genetic polymorphisms on clinical parameter variation in SCA patients.

All of the patients from this Angolan cohort had at least one CAR allele, and due to its very high prevalence in our population, it was probably difficult to highlight a statistical link. A previous study analyzed the haplotype prevalence in four Sub-Saharan African countries and was able to observe the same frequency of 92% homozygous with the CAR/CAR genotype [25]. However, they also identified five children with the AI haplotype. Another study reported an even more heterogeneous frequency of haplotype carriers with 82.2% CAR, 11.2% BEN and 6.6% SEN in Angola [26].

The CAR haplotype is typically associated with a more severe prognosis and appears to be related to greater hemolysis [27]. In our population, this was undoubtedly demonstrated by the presence of the worst clinical values among the haplotypes, namely in the hemoglobin, HbF levels, erythrocyte count, gamma ratio and LDH values. Despite the low sample population for the CAR/CAM, it was also clear from the analysis of Table 1 that it had the highest hemoglobin, erythrocyte count and HbF levels. This is not in agreement with other studies, which report an intermediate disease severity for CAM haplotypes and a less severe for the SEN and AI haplotypes [28]. However, it is important to mention that we only had heterozygotes patients for the other haplotypes, and a very low variability in our studied population.

Besides the influence of haplotypes in modulating this disease, the co-inheritance of alpha-thalassemia has also been considered an important genetic modulator, producing a milder phenotype when present. This hypothesis was already proved in this population by our research team in a previous study [29].

Regarding the BCL11A SNPs, we observed a frequency of the favorable alleles between 6.2% to 9.4% in our population, which resulted in a considerable increase in HbF levels in heterozygous and homozygous patients (Table 2). Moreover, an improvement in hemoglobin values, neutrophil count and gamma ratio was also observed. Our results are in accordance with other studies, showing that the presence of these beneficial alleles within the 14 kb intron 2 of BCL11A are strongly associated with elevated hemoglobin and HbF expression [16,27]. It is known that BCL11A acts by silencing the transcription of γ-globin genes, targeting specifically the intergenic regions, the LCR, and sequences between HBG1 and HBD [15]. 

In what concerns the combinations of haplotypes in BCL11A intron 1, as shown in Figure 4, the combination BB is the more advantageous of the BCL11A haplotypes (homozygous for CATG), providing more than double the average HbF in our cohort. Moreover, the combination AA (homozygous for TGGA) was both the most common and the one with the lowest HbF levels, accounting for 50.3% of the population. This demonstrated how four polymorphisms in an intronic region can have a major impact on the phenotype.

At HBS1L-MYB, we noticed that rs66650371, a three-base deletion, was the polymorphism with more significant associations for the clinical parameters in our cohort. This SNP was associated with increased HbF and hemoglobin levels, and it was also the only variant studied that had a significant effect on the MCV, erythrocytes, reticulocytes and white blood cell count. This variant at HBS1L-MYB had a low frequency (5%) in our population. One study in Nigeria observed a prevalence of 3%, and also demonstrated higher levels of HbF and hemoglobin when this deletion was present [30]. This deletion is located at HMIP-2A in the MYB core enhancer element, and critically affects this regulatory region, which is responsible for erythroid differentiation and indirectly controls HbF levels [17,31].

In chromosome 11, the intergenic region between the HBG1 and HBD genes contains a pseudogene (HBBP1) and a noncoding gene (BGLT3) [32]. BGLT3 is considered a developmental stage-specific lncRNA that can positively regulate γ-globin genes [33]. However, only a few research groups have tried to study this gene, and its interactions and mechanisms remain unclear. In this paper, we were able to report the first correlation between a polymorphism in the BGLT3 gene (rs7924684) and the gamma G and A globin ratio (*p* = 0.007). Alterations in this ratio is normally indicative of a molecular defect at the level of the HbF synthesis [34]. We also noticed that this variant induced a difference in the HbF levels, although this was not statistically significant (*p* = 0.053).

Besides BCL11A and HBS1L-MYB intergenic polymorphisms, the Xmn1-HBG2 site is also recognized as a major quantitative trait locus (QTL) for HbF levels, and this was demonstrated in different populations [31]. Taking this into account, we identified the rs7482144 polymorphism with a small prevalence in our cohort (1%) but with a significant increase in HbF levels and gamma ratio. One study from South-West Iran also reported these alterations on the same parameters [35]. It is known that this variant is associated with an increased expression of the HBG2 gene, and elevated synthesis of HbF and with a delayed switching from fetal to adult hemoglobin [36,37]. Interestingly, it has little effect on normal individuals, and only under conditions of hemopoietic stress does this polymorphism favor a higher HbF response [37]. 

## 5. Conclusions

This study provides a relevant contribution to the Angolan population’s genetic background, where the CAR haplotype is undoubtedly the most common HBB haplotype, and significant differences were observed in several hematological parameters in seven polymorphisms. In this work, we describe for the first time a significant association in BGLT3 gene with fetal hemoglobin.

SCA has a different clinical presentation between populations of different origins. There are several polymorphisms being discovered every day that could explain the HbF variation between different geographic regions. 

We also believe that the use of NGS approaches could expand our knowledge of SCA heterogeneity and related severity, since it allows the study of the effect of multiple variants, something very difficult to accomplish with RFLP or Sanger techniques. The results of this paper emphasize the importance of personalized healthcare for SCA patients.

## Figures and Tables

**Table 1:** Clinical and hematological variables associated with different haplotypes. The Mann–Whitney U test was used for pairwise comparisons for having or not having a particular haplotype and the Kruskal–Wallis test for multiple analysis between the four haplotype groups. Bold values denote statistical significance at the *p* < 0.05 level.

Variables	CAR/CAR			CAR/SEN			CAR/CAM			CAR/BEN			*p*-Value **
	*n* = 176			*n* = 2			*n* = 2			*n* = 11			
	Mean	SD	*p*-Value *	Mean	SD	*p*-Value *	Mean	SD	*p*-Value *	Mean	SD	*p*-Value *	
Hemoglobin (g/dL)	7.29	±0.95	0.057	7.52	±1.2	0.792	9.19	±1.15	**0.031**	7.64	±0.93	0.256	0.103
Fetal Hemoglobin (%)	5.43	±3.70	**0.013**	13.45	±0.35	**0.022**	15.10	±12.30	0.126	6.60	±3.49	0.235	**0.025**
Erythrocytes (10^12^ L)	2.92	±0.6	0.092	3.42	±1.11	0.546	4.24	±0.16	**0.030**	3.11	±0.74	0.463	0.124
MCV (fL)	77.17	±8.70	0.432	69.43	±14.88	0.403	65.26	±4.44	0.062	78.00	±8.03	0.785	0.236
MCH (pg)	25.47	±2.97	0.130	22.66	±3.91	0.212	21.80	±3.54	0.100	25.15	±2.77	0.629	0.202
White blood cells count (10^9^ L)	14.06	±4.75	0.827	12.83	±0.56	0.847	8.76	±1.41	**0.048**	14.49	±4.01	0.486	0.223
Neutrophil count (10^9^ L)	5.90	±2.34	0.764	4.11	±2.31	0.265	4.95	±1.57	0.610	6.67	±2.70	0.291	0.468
Platelet count (10^9^ L)	438.0	±174.78	0.474	433.6	±99.11	0.949	342.0	±125.87	0.396	415.3	±139.40	0.629	0.807
Reticulocyte count (%)	10.36	±4.69	0.884	8.51	±5.81	0.690	5.00	±2.81	0.059	10.68	±4.30	0.406	0.226
N° of transfusions/year	0.41	±0.55	0.169	0.00	±0.00	0.075	0.25	±0.35	0.740	0.27	±0.32	0.508	0.282
N° of hospitalizations/year	0.49	±0.51	0.524	0.25	±0.35	0.486	0.25	±0.35	0.486	0.49	±0.54	0.899	0.799
G gamma:A gamma ratio	0.53	±0.22	0.181	1.38	±0.08	0.016	0.69	±0.50	0.786	0.55	±0.19	0.707	0.108
LDH (U/L)	436.72	±154.19	0.105	742.85		0.109	300.00		0.264	344.14	±128.26	0.057	0.061

* Mann–Whitney U test; ** Kruskal–Wallis test.

**Table 2:** Clinical and hematological variables associated with different polymorphisms. Statistical analysis was performed by one-way ANOVA. Bold values with grey background denote statistical significance at the *p* < 0.05 level.

Polymorphisms			Hemoglobin (g/dL)	Fetal Hemoglobin (%)	Erythrocytes (10^12^ L)	MCV (fL)	MCH (pg)	White Blood Cells Count (10^9^ L)	Neutrophil Count (10^9^ L)	Platelet Count (10^9^ L)	Reticulocyte Count (%)	Ratio G Gamma: A Gamma
Chr2 BCL11A	rs4671393	GG (*n* = 95)	7.14 ± 0.94	4.17 ± 3.18	2.90 ± 0.62	76.46 ± 9.08	25.14 ± 3.10	13.65 ± 3.96	5.43 ± 1.92	416.48 ± 156.66	10.36 ± 4.74	0.49 ± 0.25
		GA (*n* = 79)	7.43 ± 0.90	6.71 ± 3.42	2.95 ± 0.59	77.83 ± 8.29	25.64 ± 2.83	14.44 ± 5.45	6.43 ± 2.52	455.98 ± 192.38	10.52 ± 4.52	0.58 ± 0.23
		AA (*n*= 18)	7.88 ± 1.20	8.93 ± 6.22	3.14 ± 0.76	76.73 ± 9.07	25.65 ± 3.01	14.07 ± 4.54	6.13 ± 3.07	450.05 ± 138.66	9.16 ± 4.90	0.64 ± 0.20
		*p*-value	**0.005**	**<0.001**	0.333	0.586	0.508	0.541	**0.021**	0.298	0.531	**0.006**
	rs11886868	CC (*n* = 17)	8.05 ± 1.00	9.37 ± 6.12	3.21 ± 0.71	75.69 ± 8.17	25.54 ± 3.06	13.92 ± 4.63	6.18 ± 3.16	465.26 ± 126.58	8.24 ± 3.09	0.65 ± 0.20
		CT (*n* = 78)	7.43 ± 0.90	6.69 ±3.44	2.95 ± 0.59	77.96 ± 8.26	25.68 ± 2.82	14.52 ± 5.43	6.45 ± 2.52	457.68 ± 193.02	10.55 ± 4.54	0.59 ± 0.23
		TT (*n* = 96)	7.15 ± 0.94	4.21 ± 3.19	2.91 ± 0.62	76.37 ± 9.07	25.12 ± 3.10	13.58 ± 3.99	5.41 ± 1.91	415.50 ± 156.13	10.34 ± 4.73	0.49 ± 0.25
		*p*-value	**0.001**	**<0.001**	0.166	0.399	0.451	0.426	**0.015**	0.209	0.159	**0.004**
	rs1427407	TT (*n* = 12)	8.04 ± 1.15	10.51 ± 6.74	3.11 ± 0.73	78.12 ± 9.04	26.41 ± 3.67	13.19 ± 4.86	5.57 ± 3.14	474.51 ± 168.18	8.11 ± 3.02	0.65 ± 0.29
		TG (*n* = 66)	7.49 ± 0.81	7.01 ± 3.39	3.00 ± 0.58	76.98 ± 8.41	25.41 ± 2.70	14.86 ± 5.66	6.47 ± 2.53	465.25 ± 198.56	9.94 ± 4.35	0.57 ± 0.20
		GG (*n* = 114)	7.16 ± 1.00	4.639 ± 3.22	2.90 ± 4.91	76.98 ± 8.96	25.28 ± 3.94	13.60 ± 3.94	5.63 ± 2.09	414.81 ± 151.47	10.76 ± 4.91	0.51 ± 0.25
		*p*-value	**0.003**	**<0.001**	0.336	0.909	0.457	0.183	0.071	**0.117**	0.124	0.081
	rs7557939	GG (*n* = 18)	7.88 ± 1.20	8.93 ± 6.22	3.14 ± 0.76	76.73 ± 9.07	25.65 ± 3.01	14.07 ± 4.54	6.13 ± 3.07	450.05 ± 138.66	9.16 ± 4.90	0.64 ± 0.20
		GA (*n* = 80)	7.42 ± 0.90	6.64 ± 3.45	2.95 ± 0.58	77.85 ± 8.24	25.65 ± 2.81	14.43 ± 5.41	6.45 ± 2.51	455.53 ± 191.20	10.52 ± 4.49	0.58 ± 0.23
		AA (*n* = 94)	7.15 ± 0.94	4.20 ± 3.18	2.91 ± 0.62	76.43 ± 9.12	25.13 ± 3.12	13.65 ± 3.98	5.40 ± 1.90	416.44 ± 157.50	10.36 ± 4.77	0.49 ± 0.25
		*p*-value	**0.007**	**<0.001**	0.349	0.563	0.481	0.549	**0.013**	0.304	0.531	**0.007**
Chr6 HBSIL-MYB	rs66650371	TTAC/TTAC (*n* = 182)	7.29 ± 0.96	5.50 ± 03.94	2.91 ± 0.60	77.34 ± 8.60	25.48 ± 2.91	14.19 ± 4.71	5.99 ± 2.36	437.70 ± 174.01	10.53 ± 4.65	0.53 ± 0.23
		TTAC/T (*n* = 10)	8.16 ± 0.72	8.66 ± 3.6	3.52 ± 0.82	71.79 ± 10.14	23.93 ± 3.90	10.79 ± 2.18	4.65 ± 1.60	402.75 ± 105.45	6.40 ± 2.73	0.68 ± 0.35
		*p*-value	**0.005**	**0.02**	**0.002**	**0.05**	0.11	**0.025**	0.08	0.531	**0.006**	0.81
Chr11 HBG2	rs7482144	GG (*n* = 190)	7.33 ± 0.97	5.57 ± 3.91	2.94 ± 0.62	77.13 ± 8.68	25.42 ± 2.97	14.02 ± 4.69	5.93 ± 2.34	435.90 ± 171.86	10.33 ± 4.66	0.53 ± 0.23
		GA (*n* = 2)	7.52 ± 1.19	13.45 ± 0.35	3.42 ± 1.11	69.43 ± 14.88	22.66 ± 3.91	12.83 ± 0.56	4.11 ± 2.31	433.58 ± 99.11	8.51 ± 5.81	1.38 ± 0.08
		*p*-value	0.782	**0.005**	0.284	0.216	0.193	0.721	0.275	0.985	0.584	**<0.001**
Chr11 BGLT3	rs7924684	CC (*n* = 188)	7.34 ± 0.97	5.74 ± 3.97	2.95 ± 0.63	77.04 ± 8.81	25.38 ± 2.99	14.03 ± 4.69	5.91 ± 2.36	437.22 ± 171.35	10.32 ± 4.70	0.55 ± 0.24
		CT (*n* = 4)	7.10 ± 0.82	1.85 ± 2.04	2.73 ± 0.33	77.60 ± 4.59	26.15 ± 2.38	13.30 ± 4.38	5.97 ± 1.55	372.50 ± 168.56	9.84 ± 2.62	0.23 ± 0.26
		*p*-value	0.644	0.053	0.488	0.899	0.608	0.759	0.961	0.456	0.837	**0.007**

## Data Availability

The data that support the findings of this study are available from the corresponding author (MB) upon reasonable request.
